# STAT5 induces miR-21 expression in cutaneous T cell lymphoma

**DOI:** 10.18632/oncotarget.10160

**Published:** 2016-06-18

**Authors:** Lise M. Lindahl, Simon Fredholm, Claudine Joseph, Boye Schnack Nielsen, Lars Jønson, Andreas Willerslev-Olsen, Maria Gluud, Edda Blümel, David L. Petersen, Nina Sibbesen, Tengpeng Hu, Claudia Nastasi, Thorbjørn Krejsgaard, Ditte Jæhger, Jenny L. Persson, Nigel Mongan, Mariusz A. Wasik, Ivan V. Litvinov, Denis Sasseville, Sergei B. Koralov, Charlotte M. Bonefeld, Carsten Geisler, Anders Woetmann, Elisabeth Ralfkiaer, Lars Iversen, Niels Odum

**Affiliations:** ^1^ Department of Dermatology, Aarhus University Hospital, Aarhus, Denmark; ^2^ Department of Immunology and Microbiology, University of Copenhagen, Copenhagen, Denmark; ^3^ Bioneer A/S, Hørsholm, Denmark; ^4^ Department of Molecular Medicine, Copenhagen University Hospital, Copenhagen, Denmark; ^5^ Clinical Research Center, Lund University, Malmö, Sweden; ^6^ School of Veterinary Medicine and Science, University of Nottingham, Loughborough, United Kingdom; ^7^ Department of Pathology and Laboratory Medicine, University of Pennsylvania, Philadelphia, PA, USA; ^8^ Division of Dermatology, Ottawa Hospital Research Institute, University of Ottawa, Ottawa, Ontario, Canada; ^9^ Division of Dermatology, McGill University Health Centre, Montréal, Quebec, Canada; ^10^ Department of Pathology, New York University School of Medicine, New York, NY, USA; ^11^ Department of Pathology, Copenhagen University Hospital, Copenhagen, Denmark

**Keywords:** miR-21, in situ, STAT5, IL-2, cutaneous T-cell lymphoma (CTCL)

## Abstract

In cutaneous T cell lymphomas (CTCL), miR-21 is aberrantly expressed in skin and peripheral blood and displays anti-apoptotic properties in malignant T cells. It is, however, unclear exactly which cells express miR-21 and what mechanisms regulate miR-21. Here, we demonstrate miR-21 expression *in situ* in both malignant and reactive lymphocytes as well as stromal cells. qRT-PCR analysis of 47 patients with mycosis fungoides (MF) and Sezary Syndrome (SS) confirmed an increased miR-21 expression that correlated with progressive disease. In cultured malignant T cells miR-21 expression was inhibited by Tofacitinib (CP-690550), a clinical-grade JAK3 inhibitor. Chromatin immunoprecipitation (ChIP) analysis showed direct binding of STAT5 to the miR-21 promoter. Cytokine starvation *ex vivo* triggered a decrease in miR-21 expression, whereas IL-2 induced an increased miR-21 expression in primary SS T cells and cultured cytokine-dependent SS cells (SeAx). siRNA-mediated depletion of STAT5 inhibited constitutive- and IL-2-induced miR-21 expression in cytokine-independent and dependent T cell lines, respectively. IL-15 and IL-2 were more potent than IL-21 in inducing miR-21 expression in the cytokine-dependent T cells. In conclusion, we provide first evidence that miR-21 is expressed *in situ* in CTCL skin lesions, induced by IL-2 and IL-15 cytokines, and is regulated by STAT5 in malignant T cells. Thus, our data provide novel evidence for a pathological role of IL-2Rg cytokines in promoting expression of the oncogenic miR-21 in CTCL.

## INTRODUCTION

Primary cutaneous T cell lymphomas (CTCL) represent a heterogeneous group of lymphoproliferative disorders characterized by proliferation of skin-homing malignant T cells in a chronic inflammatory environment [1]. The most prevalent clinical forms of CTCL are mycosis fungoides (MF) and the more aggressive leukemic variant, Sézary syndrome (SS) [1, 2]. The majority of the patients with MF presents with early stage disease (stage I-IIA), with approximately one third of them progressing to more advanced stages [3].

The etiology of CTCL is only partly understood, but the IL-2 receptor common gamma chain (IL-2Rgc), and its down-stream associated Janus kinases (JAKs) and Signal Transducers and Activators of Transcription (STATs) signaling pathways are frequently dysregulated and seem to play an important role in the pathogenesis and disease progression of CTCL [4]. Accordingly, the IL-2R is therapeutically targeted in CTCL by denileukin diftitox, an engineered combination of IL-2 and Diphtheria toxin (Ontac) [5]. High-affinity IL-2R's are constitutively expressed on malignant T cells. In the early stages of CTCL the IL-2Rgc-signaling cytokines IL-2, IL-4, IL-7, IL-15, and IL-21 activate STAT3 and/or STAT5, thus promoting malignant cell proliferation and survival [6–9] (and reviewed in ref. 4). While IL-2 and IL-15 appear to promote malignant cell proliferation *in vivo*, IL-21 was reported to have both pro- and anti-tumor effects [4, 10, 11]. In advanced progressive CTCL, JAK3 and STAT3/STAT5 are constitutively activated in a cytokine independent manner [10, 12] (and reviewed in ref. 4) and believed to drive a multitude of pathological events involved in skin inflammation and disease progression. In addition to the well-established role in proliferation and anti-apoptosis, the JAK3/STAT pathway drives expression of regulatory and pro-inflammatory cytokines such as IL-5, IL-9, IL-10, IL-13, IL-17, IL-22, and lymphotoxin alpha, chemokines, and factors promoting angiogenesis [13–21]. Importantly, microbes have been implicated in disease progression in CTCL [22–24], and staphylococcal enterotoxins were recently shown to trigger aberrant JAK3/STAT3 signaling in malignant T cells [25] suggesting a direct link between bacterial infection/colonization and activation of this oncogenic pathway in malignant T cells.

MicroRNAs (miRNA), non-coding single stranded RNAs of 20–25 nucleotides, constitute a novel class of gene regulators [26]. Altered regulation of miRNAs have important pathogenic implications in multiple malignancies [26]. Overexpressed miRNAs have been linked to cancer as tumor-promoting oncomiRs, while tumor-suppressive miRNAs are commonly downregulated in many cancers [26]. In CTCL miRNAs have primarily gained attention as diagnostic and prognostic markers and as regulators of malignant cell proliferation [27–32]. Recently, a three-miRNA diagnostic classifier was shown to distinguish between malignant and benign dermatoses with high accuracy [30]. Importantly, miR-155 was documented to be up-regulated in CTCL skin lesions and was associated with poor disease outcomes [32–34]. The activated form of STAT5 drives miR-155 expression, which in turn promotes proliferation of malignant T cells [27] indicating that miR-155 plays an oncomiR function in CTCL. In contrast, miR-22 is recognized as a tumor suppressor and is down-regulated in CTCL [35, 36] as well as in several other cancer types [37, 38].

miR-21 is one of the first discovered and most extensively studied miRNAs [39]. It is up-regulated in several hematological and solid-organ malignancies, displays anti-apoptotic properties [39] and promotes proliferation of CD4^+^ T cells [40, 41]. The hostgene of miR-21 (miR-21HG) is located within a coding gene *(TMEM49)* of the 17q23.2 chromosome region. Despite its intronic localization, it has its own promoter region and generates the primary transcript, pri-miR-21, which is independently regulated [42]. In contrast to several other oncomiRs, there is a good correlation between pri-miR-21 and the mature miR-21 levels, suggesting that the key regulatory step for miR-21 expression and function occurs at the level of transcription [43]. miR-21 down-regulates tumor suppressor genes [44, 45], including *PTEN* in SS [46], and may serve as a diagnostic marker for human malignancy [47]. Interestingly, miR-21 also regulates the switch from inflammation to cancer [48], an important role that is also ascribed to miR-155 [49]. Recently, miR-21 was found to be up-regulated via STAT3, involved in anti-apoptosis in malignant T cells from SS patients [12] and associated with a poor prognosis [29]. Yet, the regulation and expression of miR-21 in CTCL remains poorly understood. Here, we provide the first evidence of *in situ* miR-21 expression in CTCL skin lesions, and demonstrate that miR-21 is up-regulated in lesional skin from CTCL patients and is associated with disease progression. In mechanistic experiments, we provide the evidence that miR-21 expression is induced by the IL-2Rg-binding cytokines IL-2 and IL-15, and is up-regulated directly by STAT5 in malignant T cells. Thus, these data suggest that miR-21 and its IL-2/IL-15/STAT5 regulators play a key role in the pathogenesis of CTCL and that miR-21 may serve as a potential therapeutic target in CTCL.

## RESULTS

### Localization of miR-21 in CTCL skin lesions

**Figure 1:**
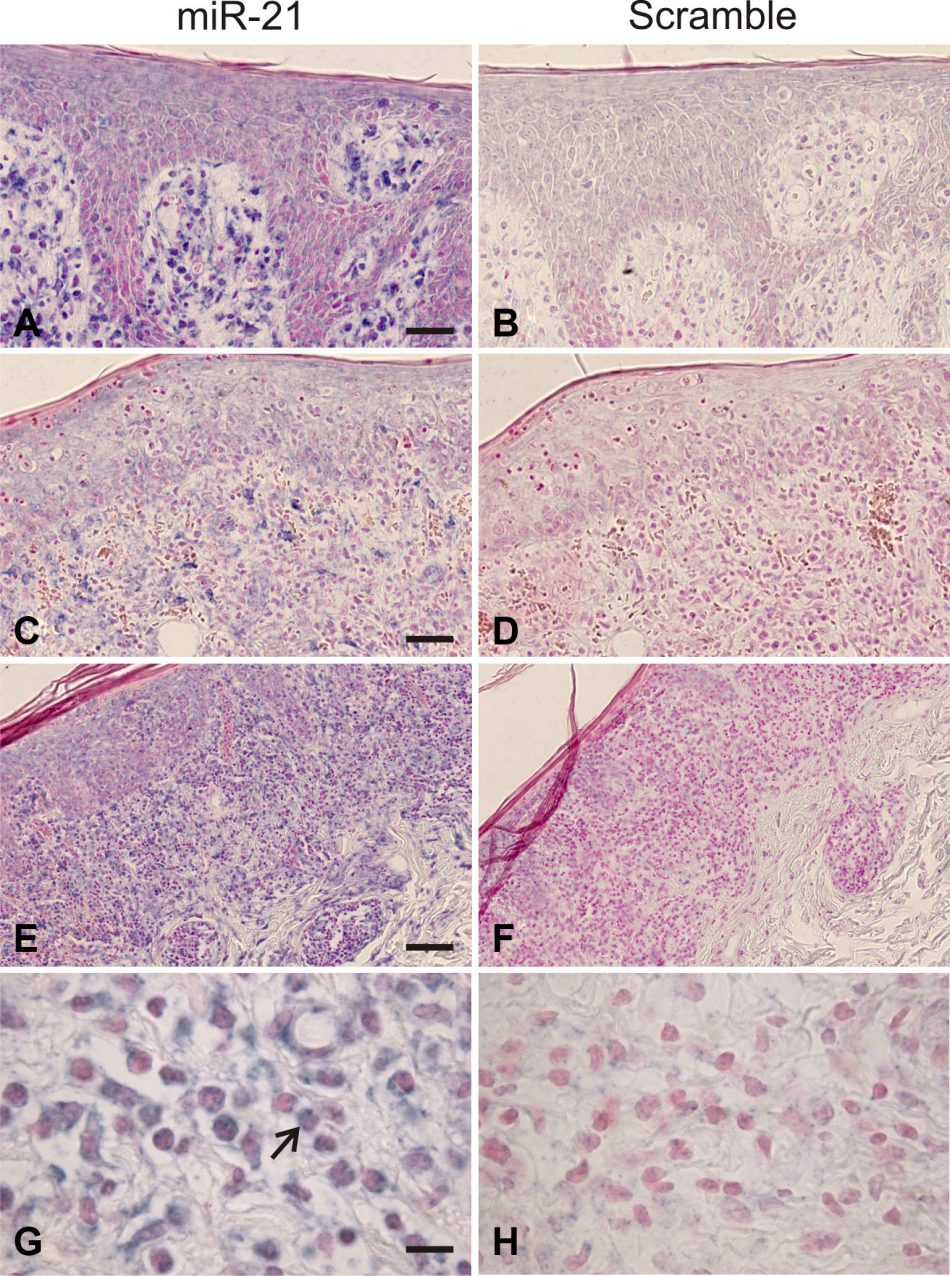
*In situ* hybridization for miR-21 in mycosis fungoides Intense miR-21 ISH signal is seen in the dermal infiltrate, and weaker miR-21 staining over epidermis, including the Pautrier's microabcesses, (**A, C, E**), whereas only background staining appeared for the scrambled control (**B, D, F, H**). Neoplastic lymphocytes (large atypical cells, example indicated by large arrow in G) and stromal cells, likely including macrophages, showed a cytoplasmatic staining for miR-21 (**G**). Similar findings were observed in other patient samples where neoplastic T-cells and stromal cells were stained for miR-21 (*N* = 10).

The miR-21 expression is increased in skin lesions and peripheral blood in patients with CTCL [12, 32]; however it is unclear which cell populations express miR-21. *In situ* hybridization analyses with a miR-21-specific probe showed a strong cytoplasmatic staining of the lymphoid infiltrate in MF skin lesions in parallel with weak background stain with the scrambled probe (examples in Figure 1). The miR-21 positive cells were localized in the epidermis, including Pautrier's microabcesses, and the dermis. Notably, both large atypical T cells with neoplastic morphology and smaller lymphoid cells resembling reactive non-malignant T cells were labeled (Figure 1H). This suggests that both malignant and non-malignant T cells express miR-21 in CTCL. Importantly, also the stromal cells including macrophages and epidermal keratinocytes stained positive supporting the notion that miR-21 is expressed by both malignant and non-malignant cells in the CTCL lesional tissue environment, as also seen in other cancers [50].

### miR-21 is aberrantly expressed in CTCL skin lesions

Next, we investigated the expression of miR-21 in lesional- and non-lesional skin from 47 CTCL patients, and 21 healthy controls. As shown in Table 1, miR-21 expression was 9-fold up-regulated in MF skin lesions as compared to healthy controls. The expression was also significantly increased (*p* = 0.027) in progressive disease as compared to patients who did not progress. These findings indicate that miR-21 is associated with disease progression, as suggested recently [31].

**Table 1:** miR-21-5p expression in skin from mycosis fungoides- and Sézary Syndrome patients and healthy individuals

MF (*N* = 42)	Fold change	*P*
Progression vs non-progression	1.5	0.028
L vs N	9.0	< 0.0001
L vs NL	5.6	< 0.0001
**SS (*N* = 5)**		
L vs N	6.3	0.003
L vs NL	1.5	0.005

MF, Mycosis fungoides; SS, Sézary syndrome; L, Lesional skin;

NL, Non-lesional skin; N, Normal skin

FOOTNOTE: Progression was defined by presence of advanced CTCL (stage IIB-IVB) at diagnosis or later, and non-progression as sustained early MF (Stage I-IIA) within the entire follow-up period (at least 5 years from onset of symptoms). Median follow-up time for the entire MF cohort was 7.8 years (range: 1.3; 32 years).

In SS patients, miR-21 is reportedly increased in the neoplastic CD4^+^ T cells circulating in peripheral blood [12]. However, miR-21 expression in skin lesions in these patients has not been previously quantified. As shown in Table 1, miR-21 was 6.3 fold increased in SS skin as compared to healthy controls, and significantly increased in lesional SS skin as compared with less affected SS skin in the same patients. These observations suggest that miR-21 could play a pathogenic role also in the cutaneous compartment of SS.

### Pri-miR-21 is constitutively expressed in malignant T cells and induced via the JAK3/STAT3/STAT5 signaling pathway

**Figure 2:**
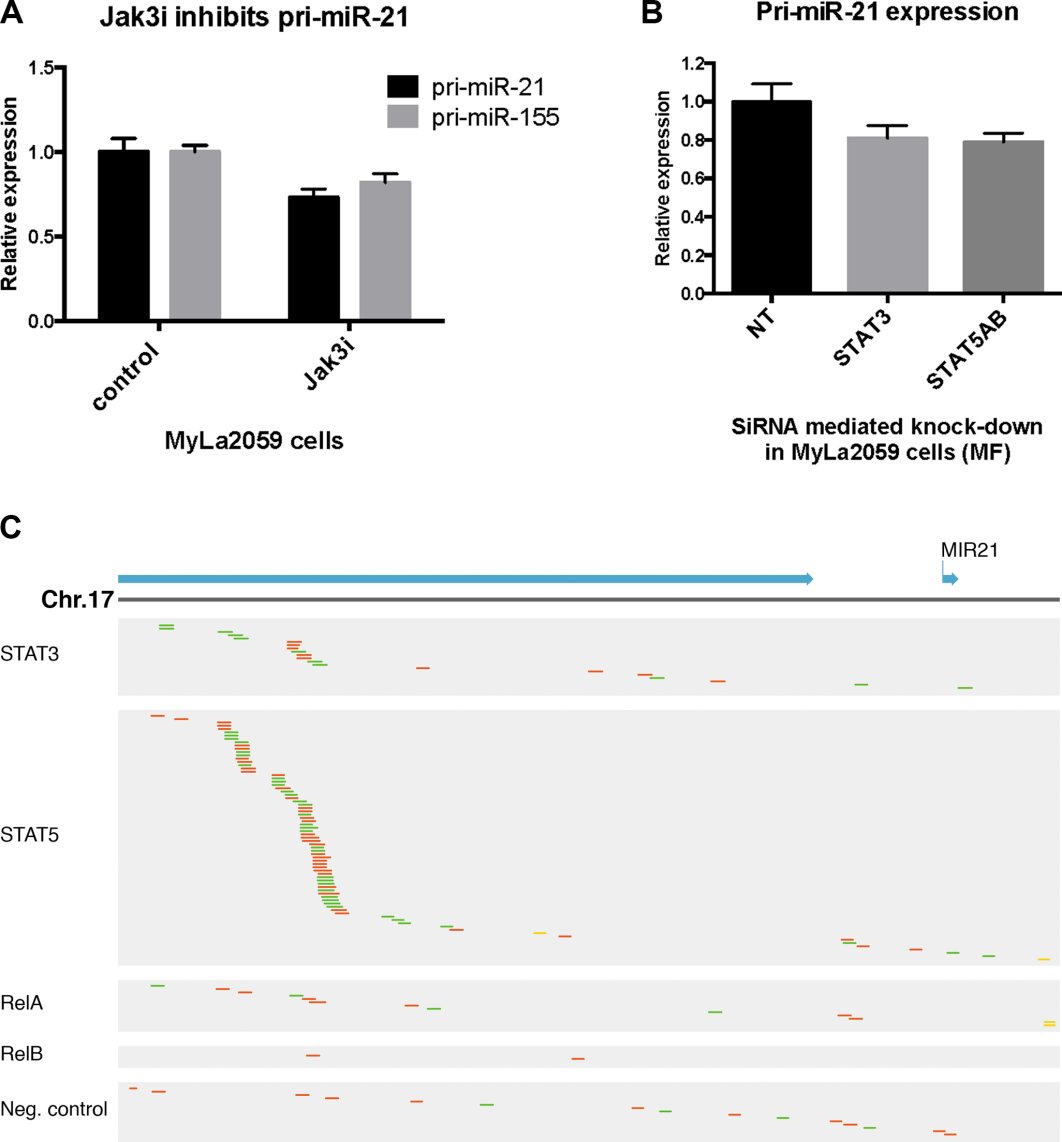
Constitutive pri-miR-21 expression is regulated via JAK3/STAT3/STAT5 in the malignant T cell line MyLa2059 (**A**) Expression of pri-miR-21 and pri-miR-155 in MyLa2059 after treatment with JAK3 inhibitor (50 μmol/L) Tofacitinib, or DMSO control for 24 hours, measured by qRT-PCR. *N* = 3. (**B**) pri-miR-21 expression after siRNA mediated knock-down of STAT3 and STAT5A and STAT5B in MyLa2059 cells as measured by qRT-PCR. (*N* = 3). (**C**) ChIP-seq reads from the miR21HG promoter region in malignant MyLa2059 cells. Reads obtained from immunoprecipitation with STAT5, STAT3, RelB, RelA and a negative control (Rabbit IgG antibody, bottom). Forward reads are indicated in green and reverse reads in red.

The JAK/STAT axis has been involved in the regulation of multiple miRNAs, including miR-21 in cancer [12, 48, 51]. To elucidate which signaling pathways drive miR-21 expression in CTCL, we examined the primary transcript of the miR-21 gene (miR-21HG/C17q23.2; 55273409–55273480), pri-miR-21, in cultured malignant T cells (MyLa2059). As shown in Figure 2A, pri-miR-21 was constitutively expressed in MyLa2059. After inhibition of JAK3 by Tofacitinib (CP-690550), pri-miR-21 expression was down-regulated, suggesting that the JAK3/STAT pathway was involved in miR-21 regulation. As expected [27], miR-155 expression was also down-regulated following JAK3 inhibition. Since JAK3 drives STAT3 and STAT5 activation in malignant T cells and both STAT proteins have been implicated in pri-miR-21 transcription in solid-organ cancers, we used siRNA-mediated depletion to address which of these STAT proteins regulated pri-miR-21 expression in malignant T cells. As shown in Figure 2B, siRNA mediated depletion of STAT3, decreased the expression of pri-miR-21. Likewise, depletion of STAT5 induced a similar decrease in pri-miR-21 expression, indicating that both STAT3 and STAT5 may be involved in the miR-21 up-regulation.

To address whether STAT3 and STAT5 directly bind to the miR-21HG promoter in malignant T cells, we performed ChIP-seq to identify transcriptional targets of STAT3 and STAT5. ChIP-seq analysis of STAT5-precipitated chromatin from malignant MyLa2059 cells yielded a strong enrichment of reads comprising a region of the miR-21HG promoter (Figure 2C, upper part, second row), confirming recent findings that STAT5 directly binds the miR-21HG and drives pri-miR-21 expression in mammary alveolar epithelium [52]. In addition, our analysis of published STAT5 ChIP-seq and ChIP-chip datasets supports STAT5 regulation of the miR-21 locus in human conventional and regulatory T cells (PMID: 24671953), CD8^+^ T cells (PMID: 25992859) and erythroleukemic cells (PMID: 24681953) (Supplementary Figure S1). As expected [53], reads for the miR-21HG promoter were also detected in chromatin precipitated with STAT3 antibody (Figure 2C, upper part, first row) but the number was lower than in STAT5-precipitated chromatin (Figure 2C, upper part, first row). The NFkB pathway is activated in malignant T cells (reviewed in [54] and as RelA and RelB drive pri-miR-21 expression in some cancers like prostate cancer [53], we addressed whether this pathway was also involved. However, only a few reads were detected in chromatin precipitated with RelA and RelB (Figure 2C, middle part), and comparable to what was seen with a control antibody (Figure 2C, lower part).

### IL-2 induces miR-21 in malignant T cells

**Figure 3:**
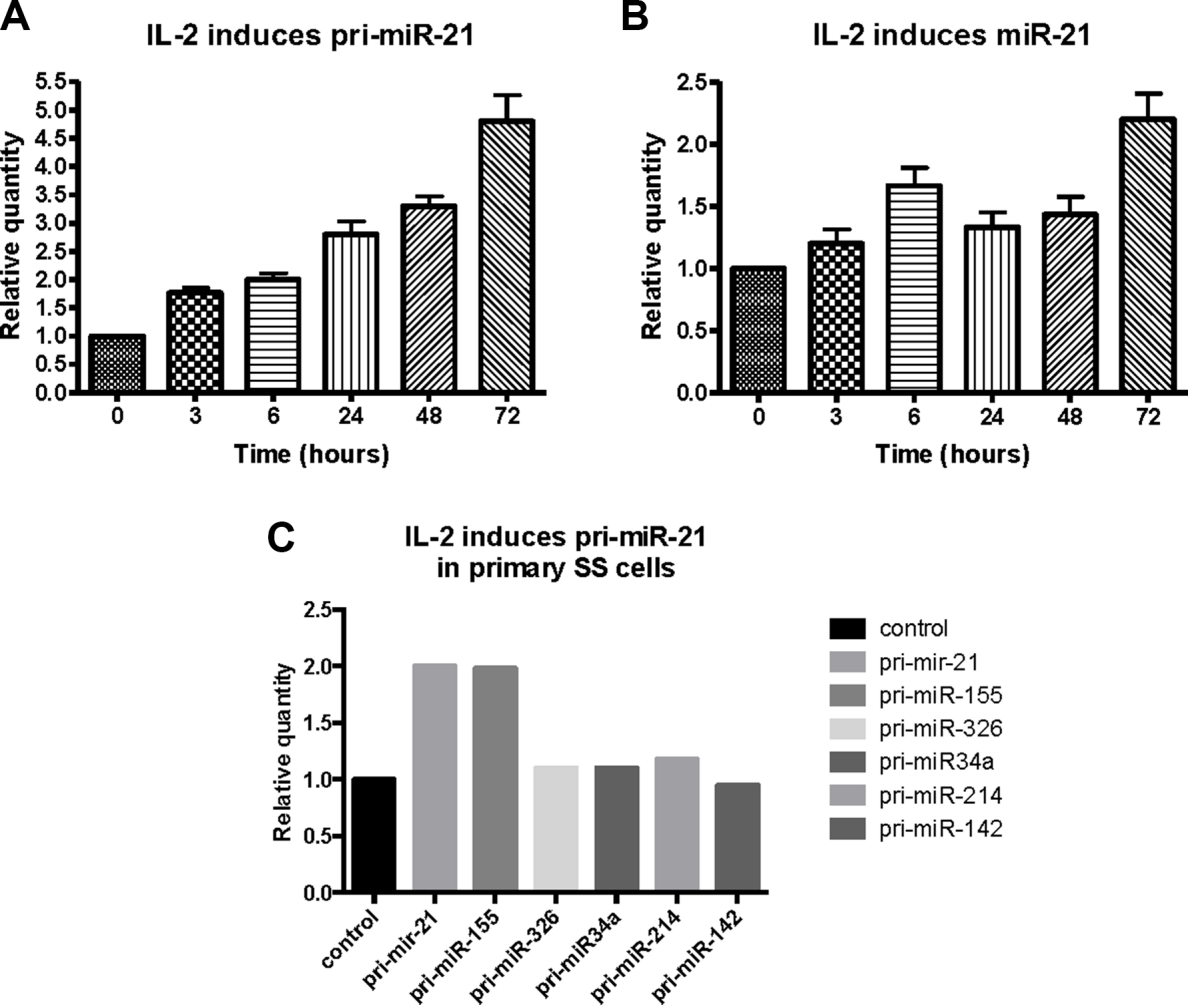
Effect of IL-2 on primary- and mature miR-21 expression in cytokine dependent T cells (SeAx) and primary Sézary T cells Expression of pri-miR-21 (**A**) and mature miR-21 (**B**) in SeAx cells cultured in the presence or absence of IL-2 for up to 72 hours, measured by qRT-PCR. *N* = 3. (**C**) Primary Sézary cells cultured with IL-2 for 16 hours followed by qRT-PCR analysis of the expression of pri-miR-21, pri-miR-155, pri-miR-326, pri-miR-34a, pri-miR-214, and pri-miR-142.

Since IL-2 activates STAT3 and STAT5 signaling via the IL-2Rg/JAK3 pathway [55], we studied whether IL-2 induced miR-21 expression in cytokine dependent T cells. We cultured malignant SS cells (SeAx) with IL-2 for different time periods, and found a time-dependent increase in pri-miR-21 expression (Figure 3A) and a similar, yet delayed, up-regulation of the mature form of miR-21 (Figure 3B). Importantly, IL-2 also induced the expression of pri-miR-21 in primary malignant T cells (Figure 3C) isolated from peripheral blood and characterized (86% CD4+ CD26-) elsewhere [25]. As expected, IL-2 signaling resulted in an enhanced expression of pri-miR-155 [27], serving here as positive control (Figure 3C). In contrast, IL-2 had little effect on other miRs (such as pri-miR-326, pri-mir-34a and pri-miR-214) previously implicated in CTCL [29, 30] (Figure 3C, right columns).

### Cytokine starvation induces a drop in miR-21 expression

**Figure 4:**
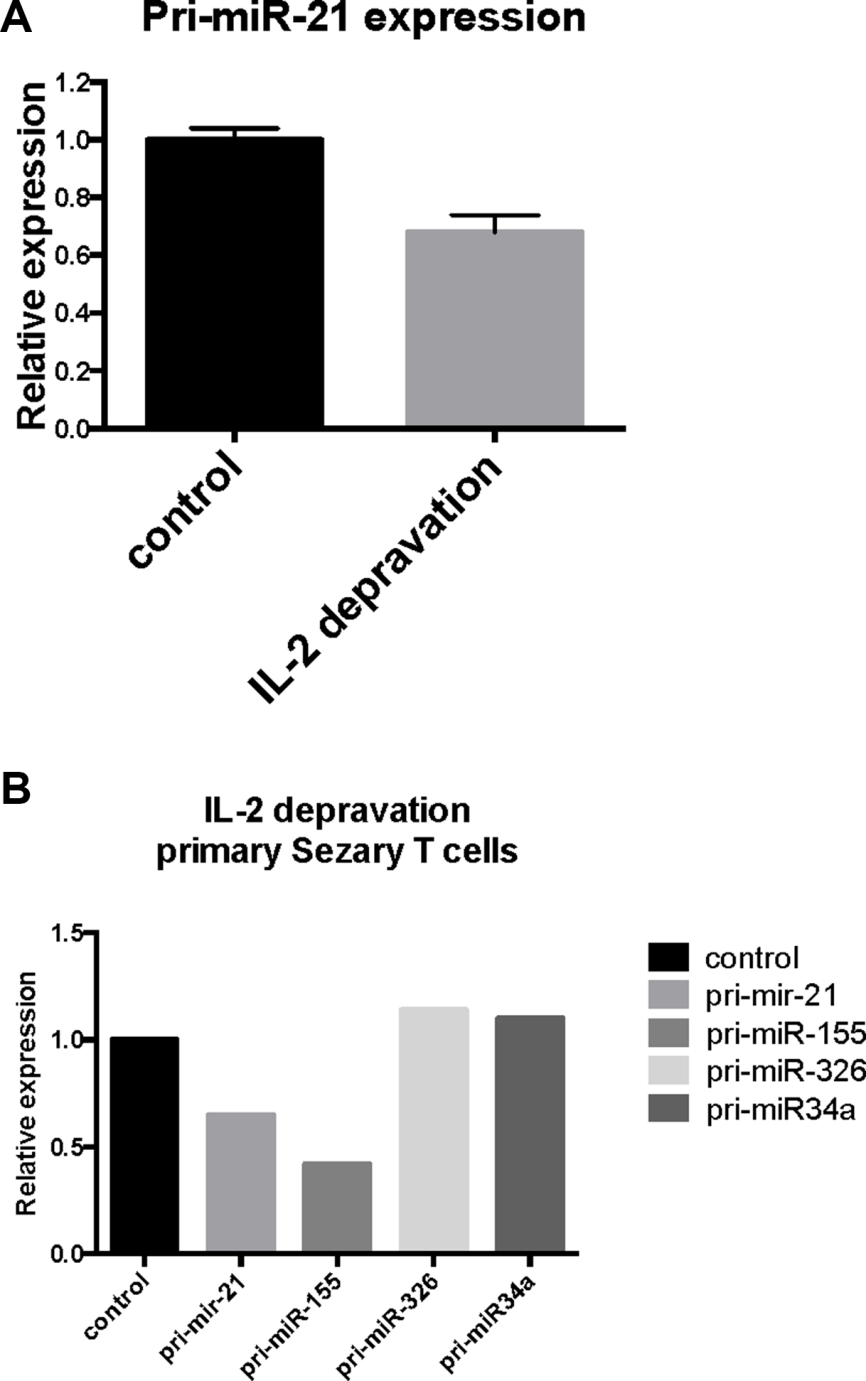
Effect of IL-2 starvation on primary- and mature miR-21 expression in cytokine-dependent T cells (SeAx) and primary Sézary T cells (**A**) pri-miR-21 expression in SeAx cells cultured with IL-2 as control and without IL-2 for 24 hours, *N* = 3. (**B**) Primary Sézary cells cultivated overnight in the presence or absence of IL-2 followed by assessment of the pri-miR-21, pri-miR-155, pri-miR-326 and pri-miR-34a expression by qRT-PCR.

To address weather IL-2 starvation of cultured SeAx cells inhibited pri-miR-21 expression, cells were cultured in the presence or absence of IL-2 for 24 hours and assayed for pri-miR-21 expression. Indeed, pri-miR-21 expression was decreased by 30% in response to IL-2 starvation (Figure 4A). Although SeAx cells depend on IL-2 for continued growth, short term starvation (16–24 h) only resulted in a slight increase in apoptosis and decrease in protein content [17]. Importantly, a similar decrease in pri-miR-21 expression was observed when primary SS cells were cultured without IL-2 (Figure 4B). As expected, pri-miR-155 expression was also decreased after IL-2 deprivation (Figure 4B). Notably, the levels of pri-miR-326 and pri-miR-34a expression were not influenced by IL-2 starvation (Figure 4B), suggesting that the decrease in pri-miR-21 (and pri-miR-155) was specific and not a result of starvation induced apoptosis. Importantly, the decrease in pri-miR-21 and pri-miR-155 but not in pri-miR-326 and pri-miR-34a following IL-2 starvation mirrored the inverse pattern of pri-miR expression following IL-2 stimulation (Figure 3C) supporting the notion above that IL-2 drives miR-21 expression in a cytokine dependent manner in malignant T cells.

### JAK3, STAT3 and STAT5 promote miR-21 expression in cytokine dependent malignant T cells

**Figure 5:**
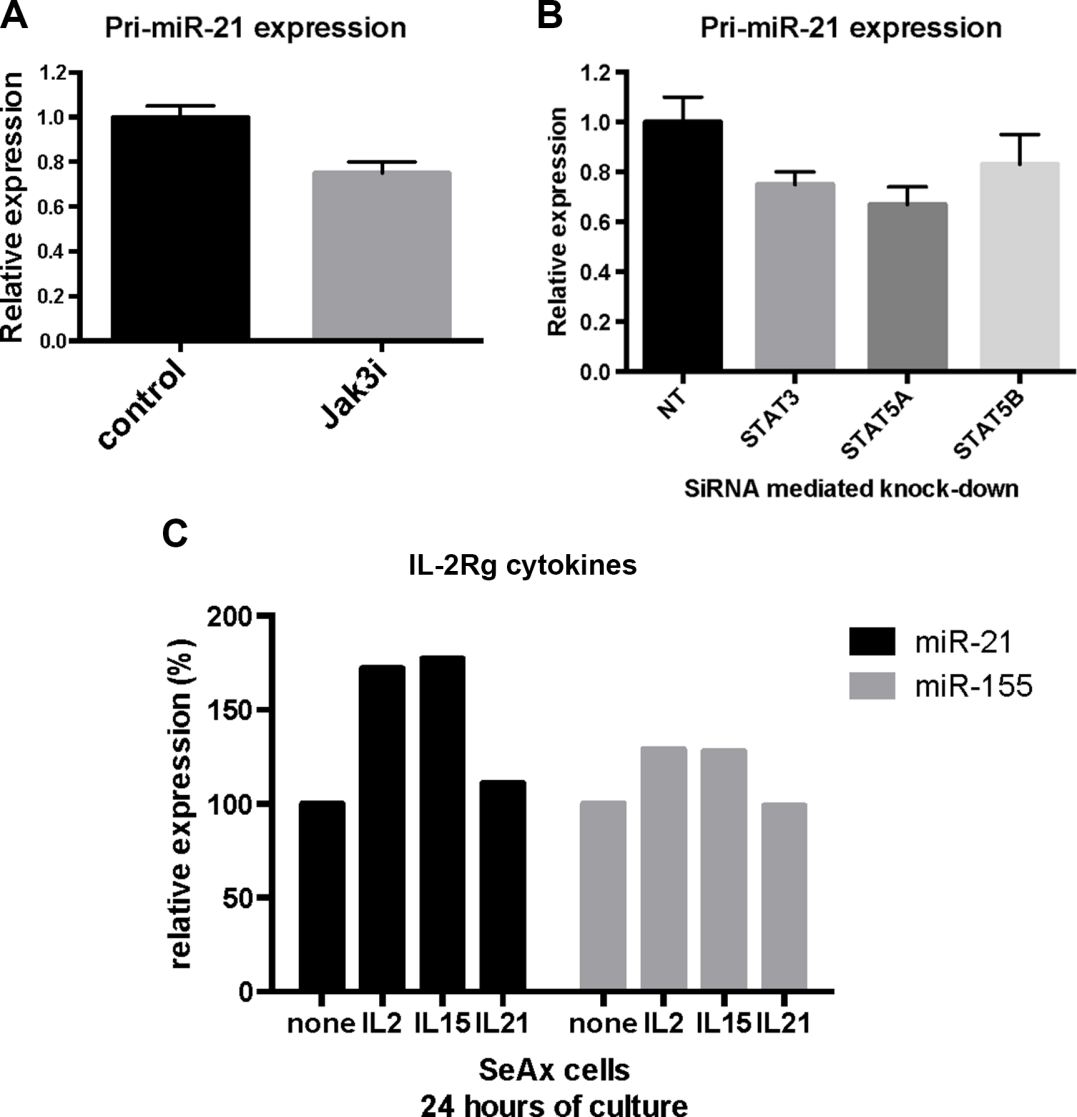
JAK3/STAT3 and STAT5 regulate miR-21 expression in cytokine dependent T cells (**A**) pri-miR-21 expression in SeAx cells cultured with IL-2 and treated with JAK3 inhibitor Tofacitinib (50 μmol/L). *N* = 5, *p* < 0.05 (Wilcoxon signed rank test). (**B**) pri-miR-21 expression after siRNA mediated knock-down of STAT3, STAT5A and STAT5B in SeAx cells cultured with IL-2. *N* = 5, *p* < 0.05 (Wilcoxon signed rank test). (**C**) Expression of miR-21 and miR-155 in SeAx cells cultured with the IL-2Rg cytokines IL-2, IL-15 and IL-21 for 24 hours. Data are shown from one experiment.

In order to extend the results described above, we examined miR-21 expression levels after siRNA-mediated depletion of JAK3, STAT3 and STAT5 in cytokine-dependent malignant T cells (SeAx) cultured with IL-2. Inhibition of JAK3 decreased the pri-miR-21 expression by 25% (Figure 5A). Similarly, inhibition of STAT3, STAT5A, and STAT5B resulted in reduction of the pri-miR-21 expression, although to a lesser degree for STAT5B, indicating that IL-2 induced miR-21 expression was, at least partly, mediated via a JAK3/STAT3/STAT5 dependent pathway (Figure 5B).

To elucidate the role of other IL-2Rg cytokines (IL-15 and IL-21) on miR-21 expression, we cultured malignant T cells (SeAx) with these cytokines for 24 hours prior to analysis of miR-21 and miR-155 expression. As shown in Figure 5C, IL-2 and IL-15 induced a clear increase in miR-21 expression, whereas IL-21 induced only a minor increase in the expression after 24 hours, providing additional evidence suggesting that STAT5, rather than STAT3, plays a key role in cytokine-mediated miR-21 induction. Similarly, IL-2 and IL-15 induced miR-155 expression, whereas IL-21 did not, confirming the previous findings that these cytokines induce STAT5-dependent miR-155 up-regulation [27]. Although IL-21 only induced a small increase in miR-21 expression after 24 hours, a substantial increase was observed after 72 hours (data not shown) confirming previous findings by Vermeer and co-workers [12, 56].

## DISCUSSION

miR-21 is well-described in cancer and believed to directly promote carcinogenesis through inhibition of tumor suppressors and enhancement of pro-oncogenic pathways, and indirectly, through induction of a tumor-promoting inflammatory environment (reviewed in ref. 49). Recent studies demonstrated that miR-21 is also implicated in the pathogenesis of CTCL. Specifically, miR-21 expression is enhanced in MF skin lesions [31, 32], and in primary SS cells [12, 29] and promotes survival of the malignant T cells. Yet, it remains poorly understood what drives its expression and exactly which cells express miR-21 within the lesional skin of CTCL. Here, we provide the first evidence that IL-2 induces miR-21 expression in primary malignant T cells and T cell lines. Thus, we show that IL-2 induced a rapid up-regulation of pri-miR-21 and a delayed up-regulation of the mature miR-21. Conversely, IL-2 deprivation triggered a rapid decrease in pri-miR-21 expression, further supporting the critical role of IL-2 in the induction of miR-21 expression in malignant T cells. Accordingly, a clinical-grade JAK3 inhibitor, Tofacitinib, strongly inhibited IL-2-induced miR-21 expression.

We also investigated downstream transcription factors involved in miR-21 expression. Our results documented that inhibition of STAT3, STAT5A, and STAT5B by siRNA-mediated gene knockdown inhibited pri-miR-21 expression, suggesting that both IL-2 induced- and spontaneous-pri-miR-21 expression was mediated through the JAK3/STAT3/STAT5 signaling pathways. Notably, other members of the family of IL-2Rg-binding cytokines previously implicated in CTCL pathogenesis, IL-15 and IL-21, also induced pri-miR21 expression. However, whereas IL-15 induced a profound up-regulation of pri-miR-21 expression comparable to that seen in IL-2-treated cells, IL-21 induced only a weak pri-miR-21 response.

Since IL-15 activates both STAT3 and STAT5 signaling, while IL-21 only activates STAT3, these findings suggest that STAT5 plays a key role in pri-miR-21 expression. Although IL-21 only induced a small increase in miR-21 expression after 24 hours, a substantial increase was observed after 72 hours (data not shown) suggesting that IL-21/STAT3 mediated miR-21 expression might involve an indirect mode of action. Our ChIP-seq analysis indicates that STAT5-immunoprecipitation significantly enriched chromatin associated with the miR-21HG promoter, whereas control antibodies did not, thus support a direct role for STAT5 in pri-miR-21 transcription. Consistent with this, other studies have demonstrated STAT5 occupancy of the miR-21 locus in distinct cellular contexts (PMID: 24671953, 25992859, PMID: 24681953). Our findings of only a modest enrichment of chromatin associated the miR-21HG promoter in STAT3-immonuprecipitats suggest that STAT3 may also regulate miR-21 expression through an indirect way. Interestingly, STAT3 was previously found to enhance miR-21 expression in some studies and repress miR-21 expression in others [12, 35, 51, 57, 58] suggesting a complex and cell context specific role of STAT3 in regard to the miR-21 expression. An extra layer of complexity arises from the observation that STAT3 is a miR-21 target itself. Thus, it is likely that STAT3 and miR-21 engage in a dynamic and complex feed-back/feed-forward regulation – a possibility, which may explain in part the discrepancies regarding the exact role of STAT3 in miR-21 expression.

Taken together, our results suggest that IL-2 and IL-15 induce pri-miR-21 expression via the JAK3/STAT5/STAT3 pathway. Thus, they provide the first mechanistic link between these two well-established CTCL growth factors and miR-21, a recently identified key player in anti-apoptosis in malignant T cells. Since advanced disease is invariably associated with increased JAK/STAT3/STAT5 activation *in situ*, our findings may also provide a direct explanation for the association between increased levels of miR-21 expression and disease progression. Of notice, our observation that the effect of IL-2 stimulation and IL-2 starvation was bigger than the effect of JAK/STAT blockage indicated that additional factors are involved in IL-2-mediated miR-21 expression. As Muppala et al [59] recently reported on Src mediated miR-21 expression in colorectal cancer cells and it is well-known that IL-2 activates Src kinases such as Lck, we speculate that Src kinases migh also be involved in IL-2 and IL-15-mediated miR-21 expression.

Although it is well established that miR-21 expression is enhanced in CTCL lesions, it remained unknown, which cell types in the skin lesion express miR-21. Now, we show that miR-21 is expressed *in situ* by malignant T cells, smaller lymphoid cells resembling reactive non-malignant T cells, and by stromal cells in CTCL skin lesions. These findings show that not only the malignant T cells, but also non-malignant T cells and stromal cells are aberrantly activated and contribute to the global miR-21 expression in lesional skin. Since miR-21 promotes inflammation and inhibits apoptosis, it is likely that the simultaneous miR-21 expression in multiple cell types is more efficient in fostering a tumor-promoting environment in the affected skin tissue. A coordinated up-regulation of miR-21 in both cancer- and stromal-cells is not unique to CTCL lesions. On the contrary, similar findings have been reported previously in solid tumors such as colorectal cancer [50]. Of notice, miR-21 has been implicated in many aspects of cancer pathobiology. For instance, miR-21 promotes genetic instability, sustained proliferation, and apoptosis evasion in cancer cells, enhances angiogenesis, and deregulates cytokine expression by macrophages and stromal cells [60]. Therefore, it is likely that a coordinated up-regulation of miR-21 by malignant and stromal cells act in synergy to promote a tumor permissive micro-environment in CTCL [39].

miR-21 is expressed *in situ* in epidermal keratinocytes and promotes keratinocyte proliferation in mice [61]. miR-21 is an important downstream component of BMP signalling in epidermal keratinocytes [61]. Moreover, miR-21 is up-regulated in hyperproliferative epidermal disorders [62] and expressed *in situ* in human epidermal keratinocytes in chronic wounds inhibiting epithelialization and wound healing in an *ex vivo* acute human skin wound model and *in vivo* in a rat wound model [63]. Induction of specific miRs inhibits cutaneous wound healing [63]. Accordingly, we propose that miR-21 expression in epidermal keratinocytes may also play a role in epidermal hyperproliferation and ulcer formation in CTCL.

In this context, our finding showing that enhanced miR-21 expression is associated with progressive disease is likely to reflect an increased expression of miR-21 in malignant T cells, as well as in non-malignant T cells and stromal cells. Indeed, malignant T cells often express IL-15 and IL-21 and may thus induce miR-21 expression by infiltrating, non-malignant T cells [4–8, 12]. Likewise, keratinocytes express IL-15 *in situ* in CTCL lesions [6] suggesting the possibility of a miR-21 promoting paracrine crosstalk between keratinocytes, malignant- and non-malignant T cells. Accordingly, we hypothesize that miR-21 has the potential of becoming an important future therapeutic target for treatment in CTCL, since miR-21 blockade is expected to inhibit not only the malignant T cells, but also the tumor-promoting environment. Several lines of evidence indicate that miR-21 is also a disease driver in other lymphomas and solid cancers. Indeed, high miR-21 expression – *in situ*, is an independent prognostic factor in DLBCL and NHL as well as solid cancers such as glioma-, pancreatic-, renal- and colorectal cancer [64–71].

In conclusion, we provide the first evidence that miR-21 expression in malignant CTCL cells is (i) induced by IL-2 and IL-15, key growth factors for malignant T cells, (ii) mediated via STAT5, and (iii) inhibited by a clinical grade JAK3 inhibitor. Furthermore, we show that miR-21 is expressed *in situ* by both malignant T cells and stromal cell and even in non-lesional skin from MF patients. Taken together, these findings support the notions that miR-21 plays a key role in the pathogenesis and progression of CTCL and may serve as a potential therapeutic target in CTCL.

## MATERIALS AND METHODS

### Patients and tissue samples

A cohort of 47 CTCL patients, 42 MF patients and 5 SS patients, and 21 healthy sex- and age-matched controls, were identified and included after informed consent was obtained. Four-millimeter punch biopsies were collected, as paired samples from the same body region, from lesional- and non-lesional MF skin, and from healthy controls. For patients with SS the biopsies were collected from lesional skin and from less affected skin from the same body region. The samples were immediately snap frozen in liquid nitrogen. The MF disease history was reviewed in medical records and patients were divided into two cohorts; a cohort of 21 MF patients displaying progressive disease and a cohort of 21 MF patients with non-progressive disease. Progression was defined by presence of advanced CTCL (stage IIB-IVB) at diagnosis or later, and non-progression as sustained early MF (Stage I-IIA) within the entire follow-up period (at least 5 years from onset of symptoms). Median follow-up time for the entire MF cohort was 7.8 years (range: 1.3; 32 years). The MF diagnosis was histologically verified for all patients, and they were staged according to the WHO-EORTC/ISCL staging system [72]. The study was conducted in accordance with the Declaration of Helsinki and approved by the Regional Ethical Committee of Region Midtjylland (M-20090102).

### *In situ* hybridization

Six μm thick tissue sections from paraffin embedded lesional MF skin biopsies were used for *in situ* hybridization as described elsewhere [50, 73]. In brief, the slides were deparaffinized and placed in a Tecan Freedom Evo automated hybridization instrument (Tecan, Männedorf, Switzerland), which was programmed to perform the following steps: proteinase-K treatment using 15 μg/mL for 8 min at 37°C, pre-hybridization in formamide-free hybridization buffer (Exiqon, Vedbæk, Denmark) at 57°C for 15 min, *in situ* hybridization with double-FAM-labeled miR-21 and scramble LNA probes (both at 40nM, Exiqon) at 57°C for 60 min, stringent washes with SSC buffers at 57°C over 33 min followed by incubation of alkaline phosphatase-conjugated anti-FAM, NBT-BCIP substrate (all from Roche, Mannheim, Germany), and finally nuclear fast red counterstain (Vector Laboratories, Burlingname, CA, USA). Finally, all slides were dehydrated and the sections mounted with Eukitt (Electron Microscopy Sciences, Hatfield, PA, USA).

### Cell lines and culture

The malignant T cell lines, MyLa2059 and SeAx, were derived from MF skin lesions and PBMC's from SS patients, respectively, and have been previously characterized [74]. Both cell lines were cultured in RPMI1640 medium (Sigma-Aldrich) supplemented with 5% penicillin/streptomycin (Sigma-Aldrich). In addition, the medium was supplemented with 10% Fetal Bovine Serum for MyLa2059 cells and 10% Human Serum for SeAx cells. The SeAx cells were cultured in the presence or absence of IL-2 (10^3^U/mL IL-2, Novartis). Peripheral blood mononuclear cells (PBMCs) were isolated from a patient diagnosed with SS using a Ficoll Gradient, Lymphoprep (Axis-Shield PoC AS), as previously described [17] and cultured as described for SeAx cells. JAK3 inhibitor, Tofacitinib (CP-690550, Pfizer), was used in experiments with MyLa2059 and SeAx cells.

### Chromatin Immunoprecipitation (ChIP) sequencing (ChIPseq)

Chromatin Immune-Precipitation followed by DNA sequencing (ChIPseq) was performed using SimpleChIP^®^ Enzymatic Chromatin IP kit (Agarose Beads) from Cell Signaling Technologies as previously described [27]. Cross-linked chromatin fragments were captured with antibodies against STAT3, STAT5, RelB, RelA, Histone H3 (positive control) or Rabbit IgG (negative control). Library construction and sequencing has been described in detail in Kopp et al. [27].

### Transient transfections with siRNA

2 × 10^6^ cells per sample were transfected with small interfering RNA (siRNA) against STAT3, STAT5A, STAT5B or non-targeting control (ON-TARGETplus SMARTpool, Thermo Scientific). Cell pellets were resuspended in 100 μL transfection solution (Ingenio Electroporation solution, Mirus) with 0.5 nmol of the respective siRNAs and transfected with an Amaxa Nucleofector (AMAXa Gmbh, Germany).

### Cell RNA extraction and qRT-PCR

Total RNA was purified from cells using miRNeasy Mini Kit (Qiagen). RNA (10ng) was transcribed into cDNA using TaqMan^®^ miRNA Reverse Transcription Kit (Applied Biosystems), and qRT-PCR was performed using TaqMan^®^ miRNA assays (Applied Biosystems) for mature miR's, according to the manufacturer's instructions and as described elsewhere [75]. U6 was used as reference gene. For quantification of pri-miR-21 expression, RNA was transcribed to cDNA using High Capacity cDNA Reverse Transcription Kit, followed by qRT-PCR performed using TaqMan^®^ Gene Expression Assays (Applied Biosystems), according to the manufacturer's instructions. GAPDH (Applied Biosystems) was included as a reference gene. The PCR amplification was performed on a Mx3005P qPCR System (Agilent Technologies) real-time cycler, using standard settings. Each experiment included three technical replicates. Results are presented as a relative quantity in comparison to the control samples based on ddCt method calculations.

### qRT-PCR panel assay

Total RNA was extracted from 4 mm skin biopsies from the cohort of 47 CTCL patients and 21 matched controls using miRNeasy Mini kit (Qiagen) according to the manufacturer's instructions. RNA (40 ng) from each patient was reverse transcribed to cDNA in 40μl reactions using universal cDNA synthesis kit (Exiqon, Vedbaek, Denmark). cDNA was diluted 100x and assayed in 10 μl PCR reactions using SYBR green mastermix in microRNA Ready-to-Use PCR, panel I and panel II, version 3R (Exiqon, http://www.exiqon.com/mirna-pcr). cDNA and SYBR Green mastermix were transferred to the qPCR panels preloaded with primers, using a pipetting robot (Exiqon, Vedbaek, Denmark). Amplification was performed in a Roche LightCycler480 in 2×384 well plates. The Roche LightCycler software was used to calculate the crossing point (Cp) value and to perform melting curve analysis for each miR in every sample. miRs detected at fewer than 5 Cps compared to the negative control or with Cps above 37, were excluded from further analysis. The PCR amplification specificity was evaluated by melting curve analysis. Reactions with a single melting point in the expected range were accepted for further analysis and reactions not reaching these criteria were omitted. Finally, reactions with PCR amplification efficacy below 1.6 were excluded from further analysis, evaluated using algorithms similar to the LinReg software.

### Statistics

The expression level of each miR in the qRT-PCR panels was normalized to the average of assays detected in all samples (*n* = 82) (average – assay Cp), which was identified as the best normalizer, using NormFinder software. Fold-change was calculated using the 2^−ΔΔ^Cp method. A Student's *t*-test was used to assess the significance of difference between expression levels in the qRT-PCR panel. In mechanistic experiments on the effect of cytokines, inhibitors and siRNAs, the Wilcoxon signed rank test was used. *p*-value less than 0.05 were considered significant and indicated in figure legends, if not otherwise specified.

## SUPPLEMENTARY MATERIALS FIGURE


