# Supplementary material for: STAT5 induces miR-21 expression in cutaneous T cell lymphoma
Source: Oncotarget. 2016 Jun 18;7(29):45730–44. doi: 10.18632/oncotarget.10160 (PMC5216756; doi:10.18632/oncotarget.10160)
Supplement: Supplementary file 1 [file oncotarget-07-45730-s001.pdf]

STAT5 induces miR-21 expression in cutaneous T cell lymphoma

Supplementary Materials

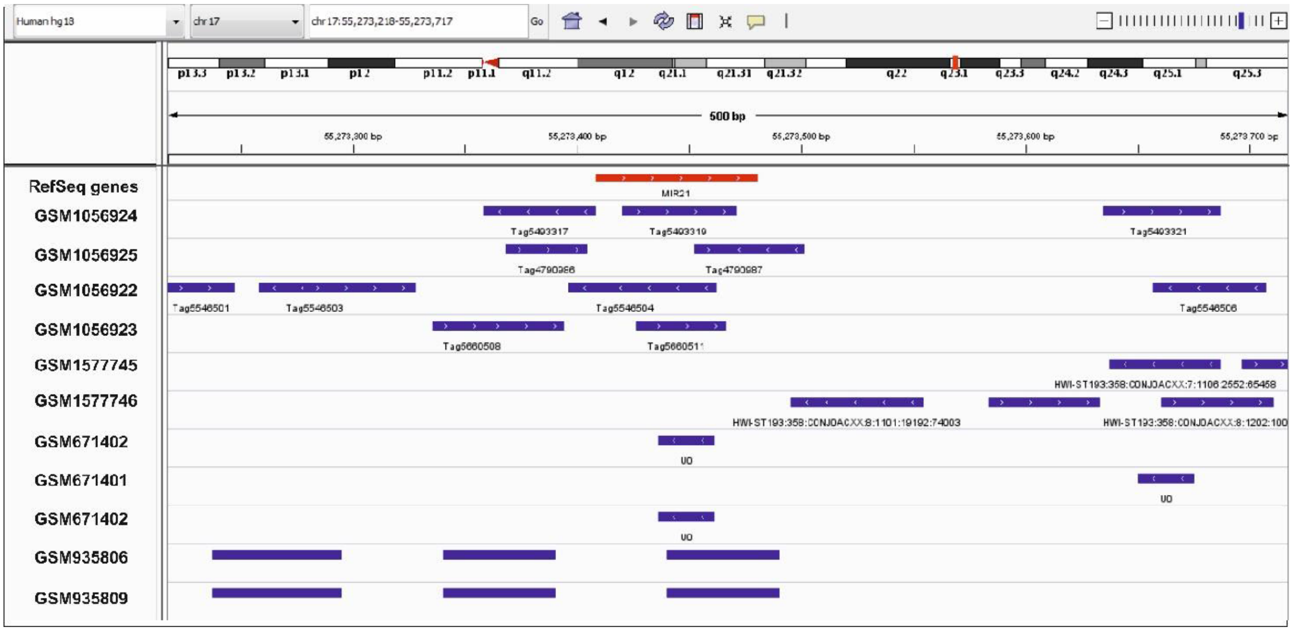

Supplementary Figure S1: The NCBI-GEO database (http://www.ncbi.nlm.nih.gov/geo/) was searched for STAT5 chromatin-immunoprecipitation (ChIP) coupled with next generation sequencing (ChIPseq) or array (ChIP-chip). Peaks (in BED) format were converted to HG18 coordinates using the UCSC-Liftover tool where necessary and visualized using the Integrated Genome Viewer. (http://www.ncbi.nlm.nih.gov/geo/). STAT5B occupancy of the miR21 locus (indicated in red) is and IL2 stimulated (GSM1577746) pre-activated CD8+ T cells; control (GSM671401) evident in human conventional T cells (GSM1056924, GSM1056925), human T regulatory cells (GSM056922, GSM1056923); control (GSM1577745) and IL2 stimulated (GSM671402) Th1 T cells and human erythroleukemic cells (GSM935806). Interestingly, STAT5B coincides with p53 at the miR21 locus in human leukemic cells (GSM935809).
